# Supplementary figures and images for: Comparative gene annotation and orthology assignments across 301 species of Drosophilidae
Source: PLoS Biol. 2026 Feb 18;24(2):e3003663. doi: 10.1371/journal.pbio.3003663 (PMC12928591; doi:10.1371/journal.pbio.3003663)

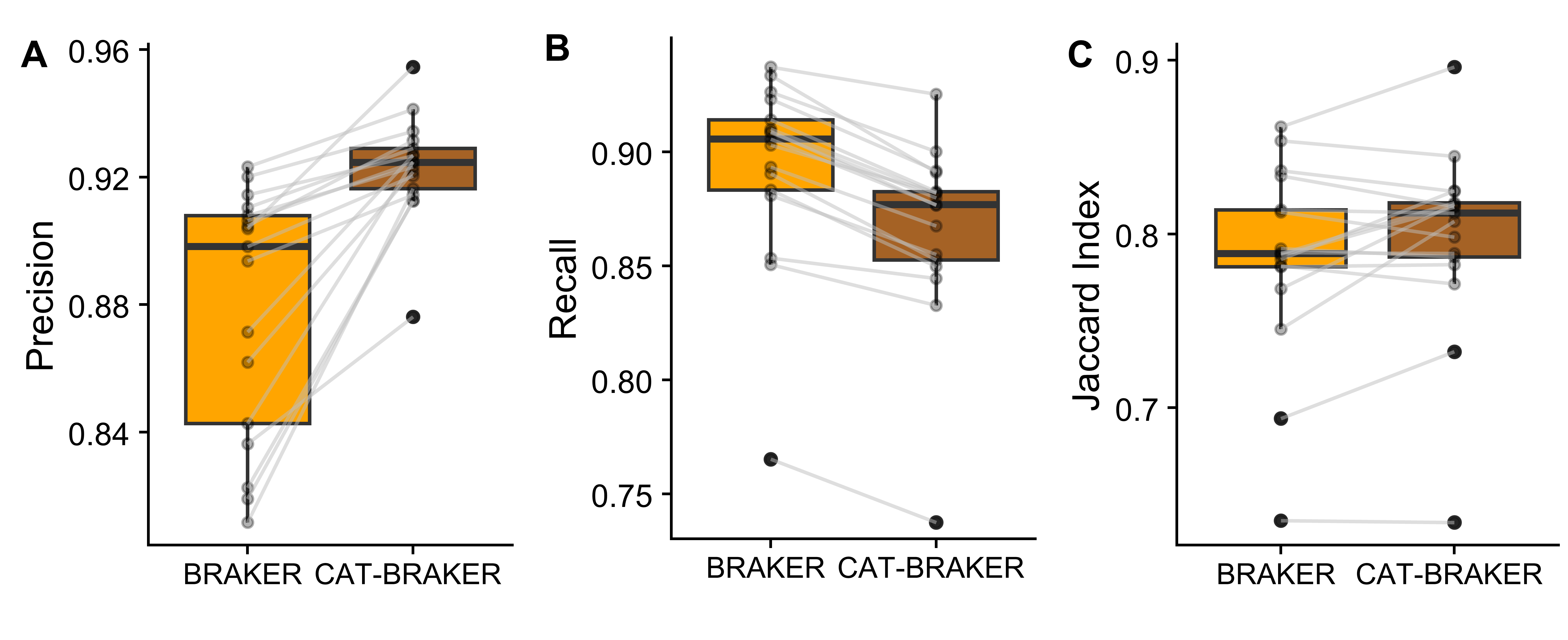

Supplement: S1 Fig — Panels show CDS-level precision (A), recall (B), and Jaccard similarity (C), quantified based on pairwise CDS overlap with RefSeq gene models. The numerical data underlying all panels are provided in S9 Table. (TIF) [file pbio.3003663.s001.tif]

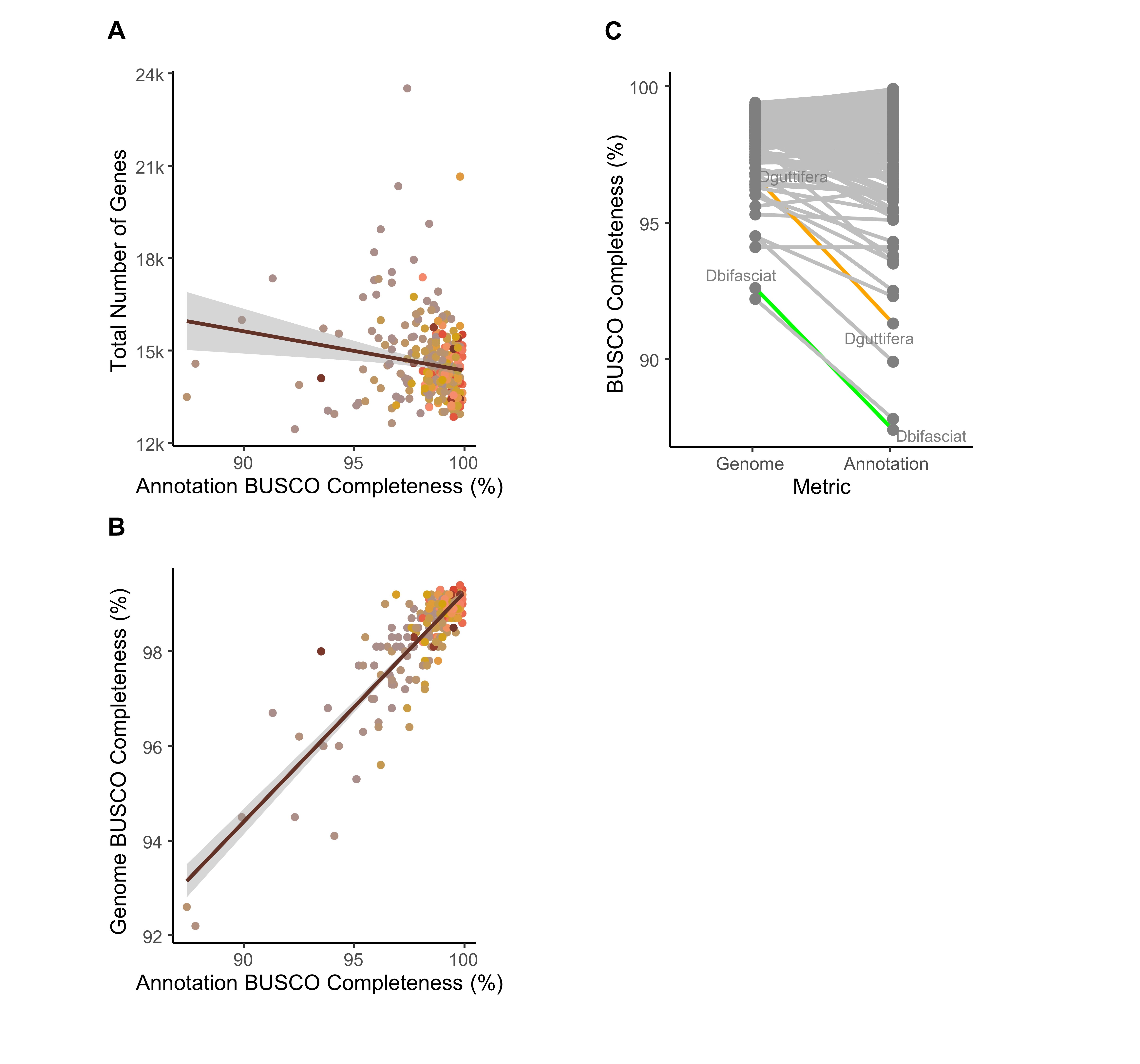

Supplement: S2 Fig — (A) Annotation-level BUSCO completeness plotted against gene number, showing a weak negative association between completeness and the number of predicted genes. (B) Parallel-axis dot plot comparing genome-level and annotated protein-level BUSCO completeness for each species. Species with less than 5% difference are colored gray. (C) Relationship between genome-level and annotated protein-level BUSCO completeness, demonstrating a strong positive correlation. Fitted lines and 95% confidence intervals in panels (A) and (C) are derived from non-phylogenetic linear models and are shown for illustrative purposes only. The numerical data underlying all panels are provided in S5 Table. (TIF) [file pbio.3003663.s002.tif]

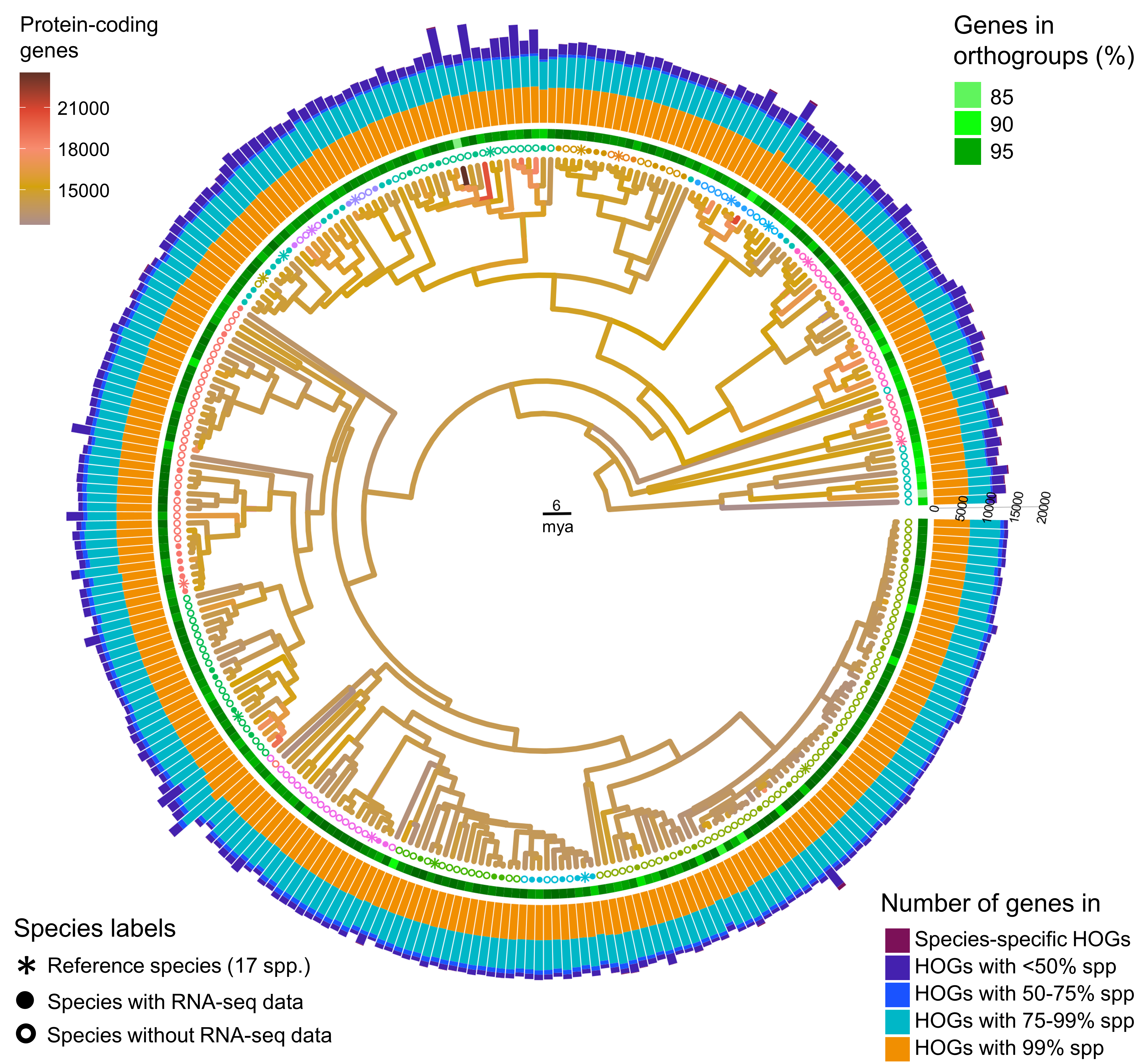

Supplement: S3 Fig — The figure shows a time-calibrated phylogenetic tree with ancestral reconstruction of protein-coding gene number mapped onto branches. Tip labels are coloured by the reference species used for comparative annotation within each clade; stars denote reference species, and filled versus open circles indicate the presence or absence of RNA-seq data, respectively. A concentric tile layer indicates the percentage of genes assigned to orthogroups for each species. The outer stacked bar layer shows the number of genes belonging to hierarchical orthologous groups (HOGs) with different levels of species representation (>99%, 75%–99%, 50%–75%, <50%), as well as species-specific HOGs. The numerical data underlying this figure is provided in S4 Table. (TIF) [file pbio.3003663.s003.tif]

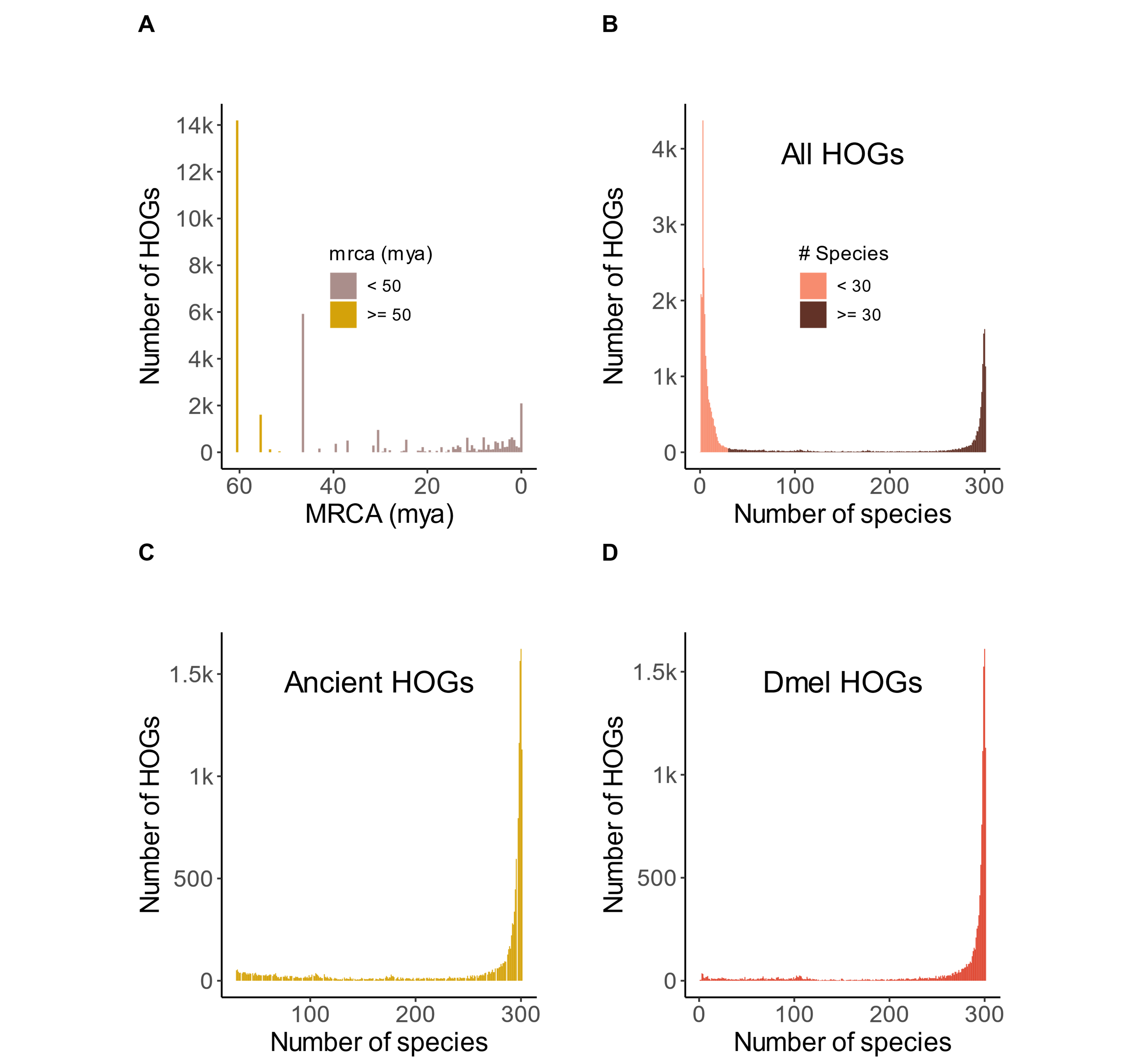

Supplement: S4 Fig — (A) Number of HOGs plotted against the estimated age of their most recent common ancestor (MRCA, in million years), reflecting the evolutionary depth of gene families. (B) Number of HOGs plotted against the number of species in which they are present. (C) Number of ancient HOGs (defined as having an MRCA ≥50 million years ago) plotted against species representation. (D) Number of HOGs containing at least one Drosophila melanogaster gene plotted against the number of species represented. This figure was generated using the HOG summary tables available from the Zenodo repository (https://doi.org/10.5281/zenodo.15016917) and the time-calibrated species phylogeny provided in S5 File. (TIF) [file pbio.3003663.s004.tif]

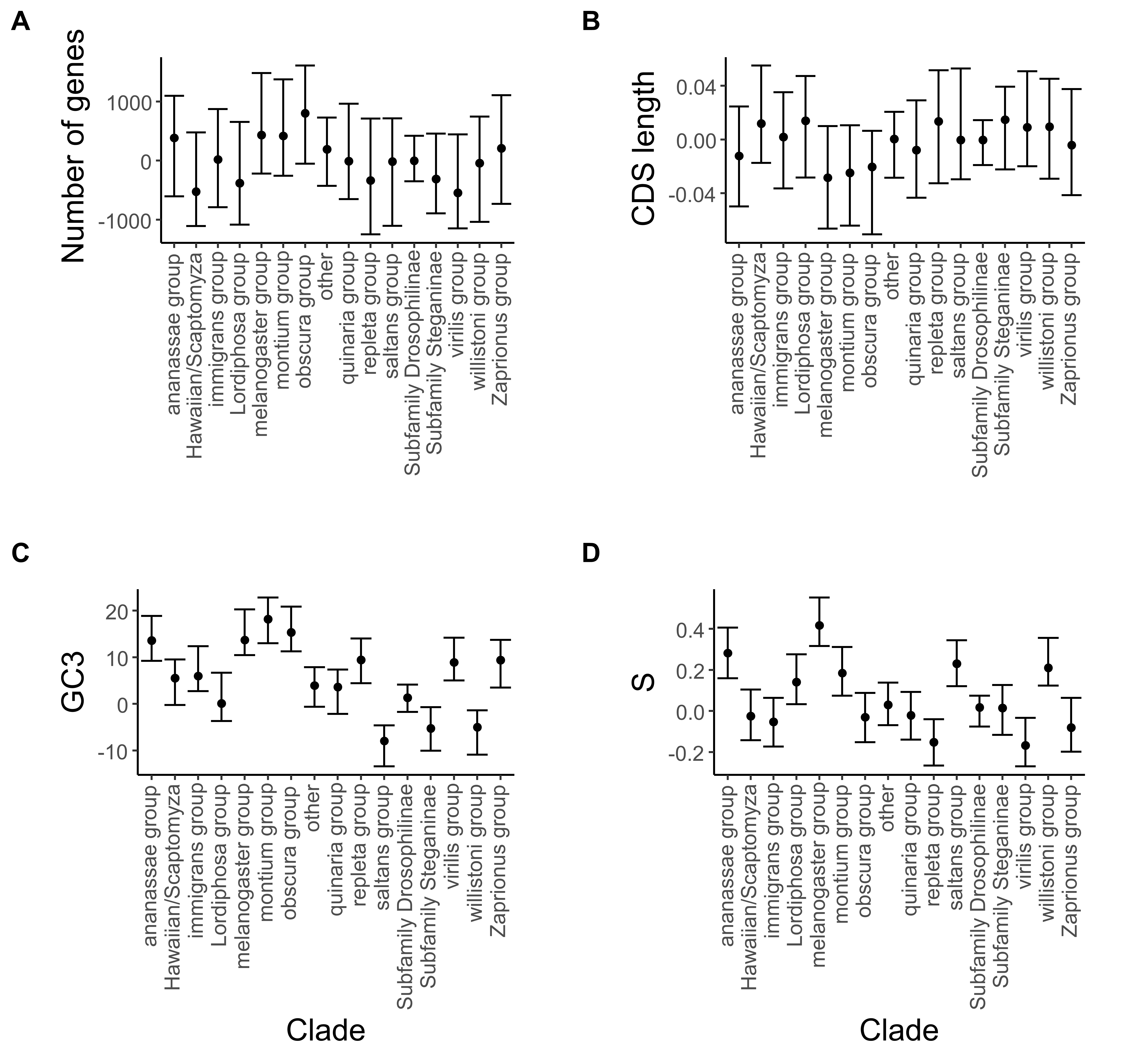

Supplement: S5 Fig — Panels show inferred values for gene number (A), mean CDS length (B), GC3 content (C), and strength of selection (S) on codon usage (D), estimated using phylogenetic mixed models. The R model objects used to generate these estimates are provided in S1 Data. (TIF) [file pbio.3003663.s005.tif]

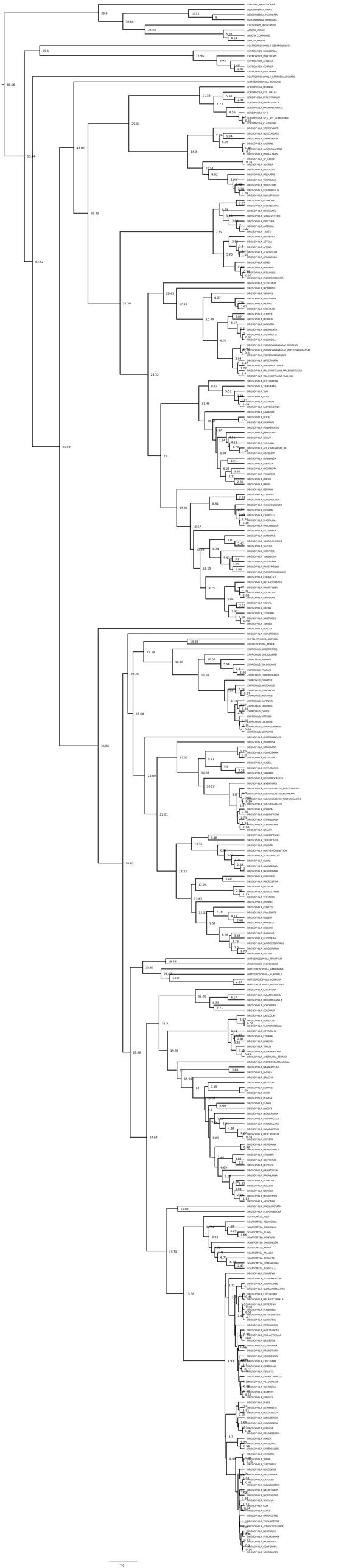

Supplement: S2 File — (PDF) [file pbio.3003663.s016.pdf]

## HOG tree

## BUSCO tree

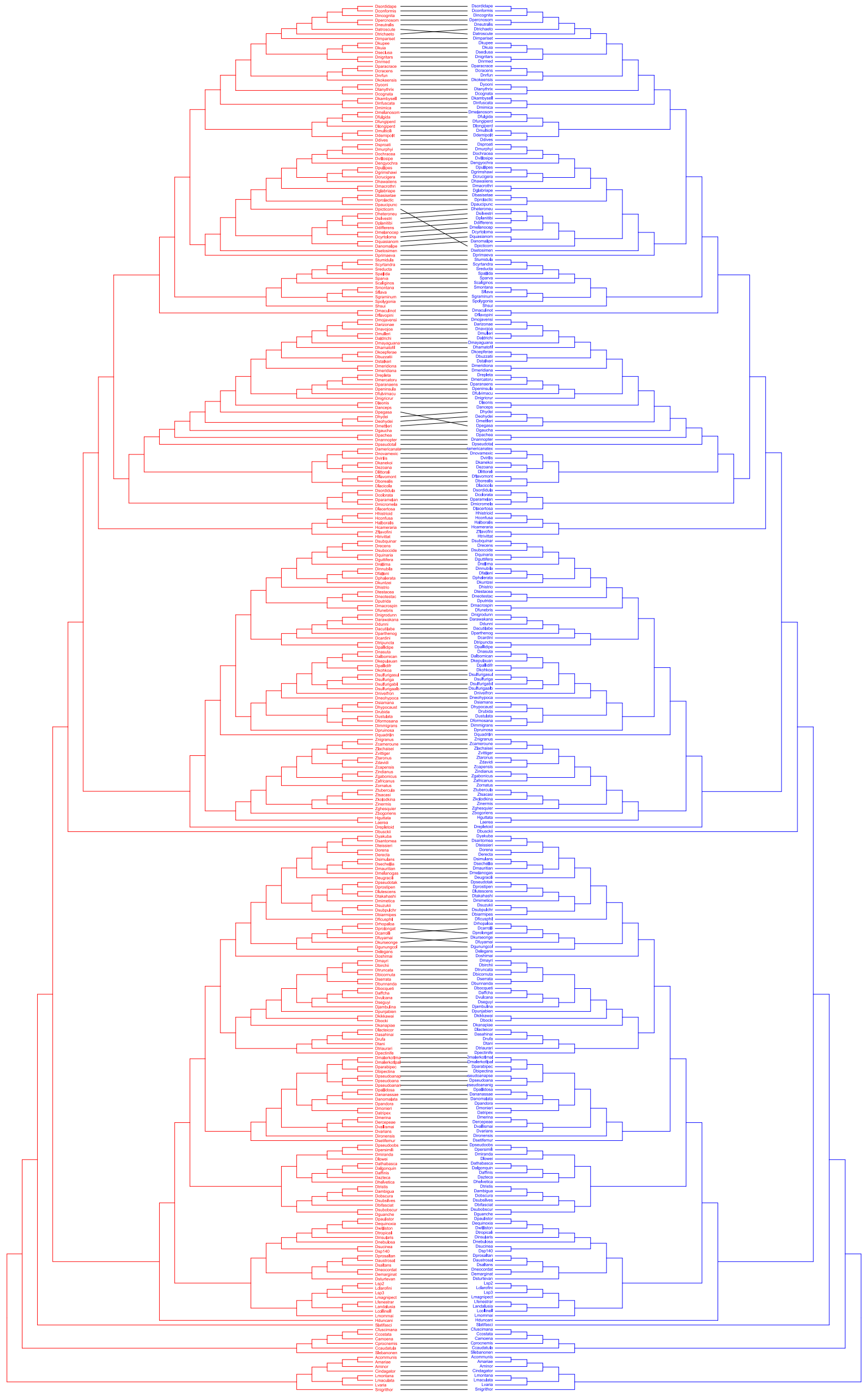

Supplement: S3 File — (PDF) [file pbio.3003663.s017.pdf]

# HOG tree

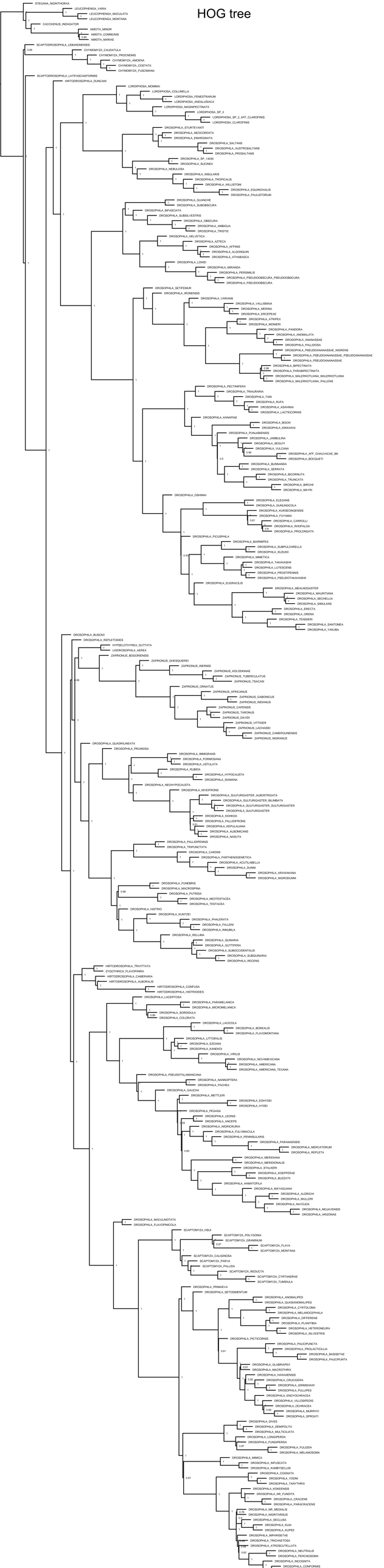

BUSCO tree

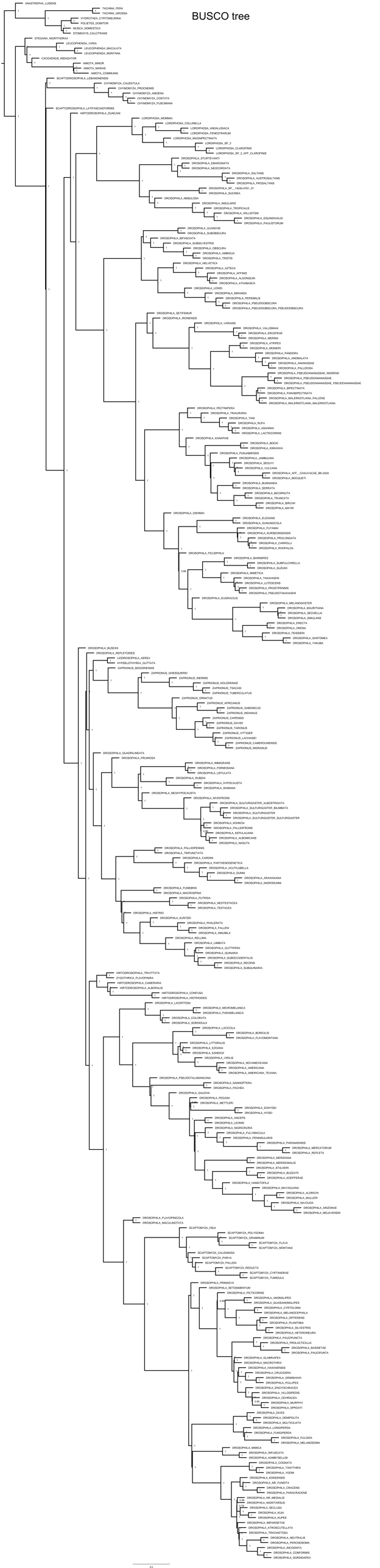

Supplement: S4 File — (PDF) [file pbio.3003663.s018.pdf]
