# Supplementary material for: Comparative gene annotation and orthology assignments across 301 species of Drosophilidae
Source: PLoS Biol. 2026 Feb 18;24(2):e3003663. doi: 10.1371/journal.pbio.3003663 (PMC12928591; doi:10.1371/journal.pbio.3003663)
Supplement: Attachment — Submitted filename: Response_to_Reviewers.docx [file pbio.3003663.s021.docx]

Response to Reviewers in blue

Reviewer #1: Dhakad et al. present the gene annotation of publicly available genome assemblies of 304 species from the family Drosophilidae, using the Comparative Annotation Toolkit (CAT) and BRAKER3, while also including publicly available RNA-seq and protein evidence in their annotation workflow. They supplement the description of their annotation resource by analysing GC and codon usage bias, as well as amino acid composition. Their extensive gene annotation, using sound methodology, in such a large number of genome assemblies is indeed a valuable resource for researchers connected to evolutionary biology and the broader Drosophila research community.

We thank the reviewer for their positive comments on the value of the resource for the drosophila research community.

Below are some specific minor comments:

Figure 1: The colours are so similar that the species/groups are hard to tell apart. It doesn't help that orders are completely different. I don't really see any substantial differences in the violin plots in Fig. 1A, so they mostly clutter the figure without providing much information.

Altogether Fig. 1A is very hard to read. Also, I wasn't able to find the alternative version of the tree in S1 File (…was expecting a figure)

In light of the reviewer’s comment and to improve clarity and readability, we have replaced the violin plots with tile layers and now summarise mean CDS length, BUSCO completeness, genome size, and contig N50. S2 File now contains a tree figure and newick files are provided separately in S5 File.

Figure 3B: The overlapping labels are partly unreadable. They should not overlap.

This has been corrected.

Figure 4: PC1 (AA) at top right should be PC2 (AA), i.e. PC1 label present twice. Also, what type of correlation is it? Pearson's r?

This has been corrected. The correlations shown in Figure 4, represent phylogenetic correlations estimated from the posterior distribution of the variance–covariance matrix of a multivariate Bayesian phylogenetic mixed model (MCMCglmm). Specifically, correlations were calculated by standardising the posterior samples of the phylogenetic covariance matrix (random = ~us(trait):label) to unit variance. Reported values correspond to posterior modes, with uncertainty assessed using 95% highest posterior density intervals. We have clarified this in the figure caption now.

S5 Figure: X-axis labels are shifted to the right and non-aligned (looks messy and does not make interpretation easier)

This has been corrected.

Line 69: "they typically don't take advantage of closely related species" - why? Isn't it up to user to select protein sequence sets from closely related species or not, using these tools?

Our intention in this sentence was not to suggest that these pipelines cannot use close homologs, but rather to highlight that they do not explicitly use whole genome alignments or conserved gene order (synteny) across multiple related genomes as part of the annotation process. We have now made this clearer in lines 69-74. We believe this revision more clearly reflects the capabilities of single genome annotation pipelines and clarifies the contrast with comparative approaches.

Line 207: Why did you prefer the CDS annotation from CAT?

We preferred CAT CDS in one-to-many or many-to-one overlap cases, as these scenarios often reflect fragmented or merged gene model predictions in single genome pipelines. The use of cross-species evidence makes CAT annotations more likely to represent coherent orthologous gene models supported by conserved exon-intron structure across species, especially for species lacking RNAseq data.

Line 384: A "but" too much?

This has been corrected.

Just a side note: Looking at the inverse relationship between mean CDS length with genome size and gene number with genome size (Fig 2, S5 Fig.) I wonder if total CDS sequence length would be quite constant across species, regardless of genome size, which might suggest more fragmented annotation in larger genomes? The authors mention annotation artefacts and assembly quality as possible reasons already though.

We thank the reviewer for this insightful suggestion. We have now tested this hypothesis by modelling total CDS length as a response variable in a phylogenetic mixed model, alongside gene number and the same set of predictors. While gene number increases strongly with genome assembly size (approx. 13 additional genes per Mbp), total CDS length increases only very weakly (approx. 13 kbp per Mbp of genome size and mean CDS length decreased by less than a bp). Thus, larger genomes did not show a proportional increase in coding capacity. This pattern indicates that the additional genes annotated in larger genomes are predominantly short and contribute little to total coding sequence, consistent with increased fragmentation of gene models or widespread gene duplication. We have clarified this interpretation in the Results section.

Reviewer #2: Summary of Manuscript

This manuscript presents a comprehensive comparative gene annotation study of 304 species in the Drosophilidae family. The authors used the Comparative Annotation Toolkit (CAT) and BRAKER3, incorporating RNA-seq and protein evidence, to generate protein-coding gene annotations. They employed a phylogenetic approach to improve consistency and accuracy in annotations and orthology assignments.

Using phylogenetic mixed-model analysis, they found moderate phylogenetic heritability for gene number and CDS length, suggesting that both evolutionary history and species-specific factors influence these traits. To demonstrate the utility of their annotations, they investigated codon usage bias and amino acid composition across Drosophilidae. Their findings indicate that codon usage correlates with overall GC content and evolves slowly, but is also strongly influenced by selection.

The authors pitch this effort as part of an ongoing community effort to improve genomic resources for Drosophilidae, aiming to facilitate research in comparative genomics, including studies on new gene origins, genome size variation, and evolutionary forces shaping gene content and structure.

My impressions

I think it's important to note that this is an expansive, time-consuming project with a very large scope. It is a well-considered and self-evidently time-consuming project. I congratulate and thank the authors for their contributions. It's equally important to note that this work also aspires to be a resource that will be widely used by the community. In light of that, I feel that, on its own terms, this manuscript is suitable in scope and impact for PLoS Biology. What remains for discussion is whether it achieves its goals to an appropriate level.

We thank the reviewer for their positive comments on the scale of the work and its potential value to the drosophila research community

I am very enthusiastic about this manuscript and I am hopeful that it will lead to a resource that will accelerate research for the target community. However, I do have a substantial number of substantive constructive criticisms. In my view, these criticisms must be addressed to ensure the manuscript lives up to its ambitious goals. The current state of the work does not accomplish this. The reason I am being so critical in this area is that I foresee that this manuscript, if published in its current form, will become the de facto annotation resource used for Drosophila until something better comes along, which may be for quite a while. Due to the high integrity of the authors themselves, it is clear that using such a resource will be replete with many caveats on a genome-to-genome basis. In my assessment, this substantially limits its utility. Many users will either shy away from using it in areas where it is clearly a bit rougher, or even worse, may use it with less sophistication and care than is clearly warranted for many genomes and will come to erroneous conclusions as a result.

I think the primary problem is quite straightforward: heterogeneity in genome quality (i.e., assembly quality, gene evidence presence/absence, transcriptome quality, etc). I think the variation in quality is very often cited in the authors' own discussion as a reason for some apparently anomalous observations. To be clear, this variation in quality is a feature of the existing resources the authors are relying on and not in their direct control.

My suggestions are a only a little less simple:

1) exclude more dubious genomes

2) validate the genomes that they maintain as meeting a base level of quality higher than what they currently provide

While this seems fairly vague, I think the authors will know they've achieved it when they aren't focusing so much on explaining anomolous observations in their resource by referring to poor assembly quality.

* I think it's important that QC metrics accompany all genomes that the authors opt to use. The authors have done several, but not all of these:

- heterozygosity/ploidy (GenomeScope)

- Base level quality scores / phred / QV (Merqury)

- Contamination

- Contiguity (Contig N and L stats, probably more than just N50)

- Completeness/Level of Duplication (e.g., all BUSCO stats, particularly don't hide high duplication by only showing the total complete category)

The reviewer raises a number of very important points. As explained in detail below, in response, we have now re-run our analyses excluding three potentially problematic genomes of *Drosophila pseudoobscura pseudoobscura* (GCA_000001765.3), *Drosophila americana* (GCA_030788265.1), and *Drosophila paucipuncta* (miss-spelled as “*D. paucipunta*” in previous annotation set, so there were two genomes of the same species) and also run all of our analyses using an additional second subset of the 215 best-assembled genomes that meet the following criteria: BUSCO completeness ≥97%, contig N50 ≥2 Mbp, estimated OMArk contamination ≤5%, and assemblies generated using long-read sequencing technologies

We now present (and make available) both datasets. As the analyses do not find any substantial differences in gene-level metrics, we focus on the more comprehensive dataset, while making the higher-threshold dataset available in parallel, and highlighting similarities and differences where appropriate.

We wish to emphasise that for many users, who wish to know about homologues of a single gene in multiple species (e.g. sequence evolution, alignment, fold predictions, conserved regions) poorly-assembled genomes are unlikely to be a major problem: occasional absent sequences are simply missing data, but the more sequences available the better for all other purposes.

* Manual QC

When the genomes come from sources where the QC isn't understood or isn't very comprehensive, it is a good idea to do these for this resource.

We now include more detailed quality metrics for the species used. However, given that the impact of excluding the most problematic genomes appears very small – especially for the many use cases that likely focus on single genes – we have chosen not to perform a more manual curation on all genomes. We regret that such an undertaking is simply beyond the resources that we have available.

For example, in many assemblies, there are idiosyncratic reasons why some important procedures may not be adhered to. Some examples come to mind. In D. miranda, the authors include the Neo-X and the Neo-Y, which is very young and inflates the number of apparent duplicates.

We now discuss the important case of the very new *D. miranda* neo-Y in more detail within the manuscript.

In some genomes, some authors may include alternate haplotypes without communicating this to GenBank, and as a result, known althaps are mixed in with the regular genome, also artificially inflating duplicates. To combat this, it would useful to ensure that all genomes without a clearly articulated QC procedure (i.e., Kim et al. 2024 is more clearly articulated than many other genomes) should undergo at least de-duplication and contamination purging to be consistent.

If alternative haplotype assemblies were generated, but were erroneously included and treated as a primary assembly, then a very large proportion of genes would appear duplicated (i.e. all those on the secondary haplotype) – rather than a small proportion (as would arise from miss-assembly, or true duplication). This is not generally true for our dataset; that is, there are no genomes (except those mentioned above) with large-scale duplication that could be attributable to the systematic or inclusion of secondary genome-wide haplotypes. This perhaps could be a problem for poorer assemblies with very short contig N50, in which only a few secondary-haplotype contigs might be erroneously retained, but (as our new analyses show) the effect appears small, e.g. being a short-read only assembly adds around 1000 genes in the 301 species dataset, and adding a Mbp to the N50 of an assembly only reduced the number of genes in the assembly by 13 (p = 0.051).

* Y chromosomes sometimes throw monkey wrenches into things, especially if they are new or otherwise have interesting biology. It would be really useful to account for this when known and practicable, though I'm sympathetic that doing this generically is way out of scope. I have in mind neo-Y systems like D. miranda and D. albomicans.

* The authors seem to be following a rubric for discussion of large patterns that goes something like this: whenever something out of the ordinary crops up in (sparse HOGs, e.g.), the idea of annotation error or misassembly is offered as a potential explanation. I'd much rather see a dataset the authors are sufficiently confident in that unusual observations offer legitimately interesting biology because the most likely artifacts have been removed. It's far less interesting for the authors to be constantly reminding readers that the observations they are bringing to our attention may not be biologically meaningful due to heterogeneous assembly/annotation quality. I'm very supportive of the authors bringing such limitations to the reader's attention. But at the same time, if there are that many caveats about this resource, is it really ready to serve as a resource yet? If the caveats loom this large, then using it will be an exercise in caveat emptor rather than a convenient resource.

We firmly believe that, as a resource paper, it is essential that the resource is made available and that authors are free to include or exclude whichever species they wish. Inclusion should be the users informed decision, not ours, as what species should, or should not be included, depends entirely on the analysis to be undertaken. An author interested in knowing which is the most conserved region of a specific gene has very different requirements to one who is interested in gene turnover. Someone in the former category, such as a molecular lab, may not be well placed to annotate 300 genomes to find their gene. Someone in the latter category will already be aware of the issues. We believe that it is much better that the data are made available in the best form that we are able to make, than to let it languish for another decade (as has happened for many of the genomes sequenced since 2007).

* Alternate haplotypes, duplicates, and neo-sex chromosomes can all lead to copies of the same basic gene model with genetic variation between them. This is often simply interpreted as duplication, when, in fact, there are three separate processes that lead to this. The authors should incorporate this basic fact about assessing assemblies into account.

* RefSeq usage: There are two issues I see.

1. The usage of RefSeq is hard to understand:

L162 "We selected 37 ‘reference’ species for lift-over annotations based on the completeness and

quality of their genomes, as indicated by RefSeq annotations [36]."

L184 "To perform the genome annotations, we first prepared the reference annotations and extrinsic

‘hints’ for use in CAT [22]. RefSeq annotation files were converted using the “convert_ncbi_gff3”

script provided by CAT, and the resulting GFF3 files were validated with the “validate_gff3”

script to ensure compatibility. We then employed three modes of AUGUSTUS [16] in CAT: two

based on transMap projections (AugustusTM/R) that project annotations from reference

genomes onto target genomes, and one using ab-initio and comparative gene predictions

(AugustusCGP) guided by extrinsic hints [57]."

I don't understand whether RefSeq annotations actually appear in your resource as the annotation being used or whether they merely feed the annotation software as hints that themselves lead to a separate annotations. Please clarify.

We thank the reviewer for highlighting this ambiguity. Of the 37 candidate species with RefSeq annotations, 17 were selected as reference annotations and used as inputs to CAT for annotating other genomes. The final annotation resource released in this study consists of CAT-derived annotations except for these 17 species. We have now made this clearer in lines 178-194.

2. The authors are missing an important feature of using a well-studied genus like Drosophila. There are several high-quality assembly/annotation combinations that could be used to validate the quality of the annotation procedure. For example, the authors could compare, for example, Drosophila innubila's high quality RefSeq annotation to a D. innubila annotation derived from the authors' procedure to evaluate their approach against a gold-standard in a high quality assembly. This could be repeated for selected high quality annotations. If the authors are already directly using the RefSeq annotations, then obviously this wouldn't be possible and maybe an approach that annotates these genomes without recycling the RefSeq data directly could work. If the authors *ARE* simply lumping in RefSeq annotations with their own bespoke annotations, I would expect a more detailed discussion of comparing annotations derived from different annotation pipelines.

We agree with the reviewer that high-quality RefSeq annotations provide an opportunity for validation. As the reviewer suspected, we compared our CAT-derived annotations against RefSeq and BRAKER3 annotations for selected non-*D. melanogaster* species, including *Drosophila innubila*. These comparisons are presented in the Results section (lines 399-411).

* L190 "We used Comparative Gene Prediction (CGP) parameters trained on 12 well annotated Drosophila species from the Drosophila 12 Genomes Project, based on exon and intron scoring"

The parameters estimated from that 2016 study are themselves based on the original 12 genomes data, which is frankly terrible. This is not a criticism of the paper, but it is from data published in 2007 and collected before that. If these CGP parameters play an important role in annotation, I suggest justifying or removing them. Notably, non-melanogaster genome assembly quality in 2016 and before is really low. It wasn't until assemblies inspired by studies like Kim et al. 2014 (10.1038/sdata.2014.45) started to change that. And it was definitely after the Konig et al. 2106 studty [57].

We thank the reviewer for raising this important point and agree that early non-melanogaster genome assemblies were more fragmented than those currently available. However, the CGP parameters used in CAT do not derive directly from genome-wide annotations of those assemblies. Instead, these parameters were trained using a curated set of 320 single gene sequences from *Drosophila melanogaster*, rather than relying on genome annotations from fragmented assemblies. As a result, these parameters primarily capture conserved splice-site signals, exon length distributions, and intron characteristics that are largely independent of assembly contiguity and remain stable across Drosophila species. Moreover, within the CAT pipeline, AUGUSTUS-CGP is only one component contributing to the final gene models. CGP predictions are informed by multiple complementary sources of evidence, including whole-genome alignments and transMap projections from high-quality reference annotations, as well as ab initio predictions guided by extrinsic hints. The CGP parameters therefore do not act in isolation, but rather provide a probabilistic framework that is constrained and refined by comparative and transcript-supported evidence. Consequently, inaccuracies in any single component are unlikely to dominate the final annotation.

* L277 "To assess the factors influencing gene number and CDS length across drosophilid species, we fitted a multivariate phylogenetic mixed model using MCMCglmm [65]. Our model included gene number and mean CDS length as response variables, allowing us to analyze their (co-)variation with respect to predictors such as distance from reference, RNAseq availability, status as a liftover reference, assembled genome size, and assembly scaffold N50."

It seems like Scaffold N50 is a poor co-factor compared to contig N50. A garbage assembly can be scaffolded into one with a long scaffold N50 with relative ease, and yet the underlying contigs will be a poor representation of the genome. Moreover, scaffolding short contigs is error-prone compared to scaffolding long contigs. And in any event, do all assemblies even have scoffold N50s? Also, it seems like many assemblies are likely from Illumina alone. I think adding something that captures whether or not long reads were used would explain a lot of the variance.

* L123 Minimum N50 of 50kb is way too low in my opinion. I was surprised that a higher threshold wasn't used. I personally consider Drosophila genomes with N50s below 10Mb to be suspect, though I could live with a 1Mb threshold. I would also like to see BUSCOs above 95%.

* L496. No one metric is a reliable proxy for assembly quality, and certainly not N50. Indeed, "assembly quality" is a broad term that encompasses both accuracy of bases and accuaracy of the reconstruction of the structure. It can also include contiguity, of course. I'd like the authors to acknowledge that contiguity isn't the only salient metric of assembly quality.

* L499-520. This is very descriptive and speculative. The authors are generating a lot of hypotheses with their speculations. Why not test some of them?

We agree with the reviewer that assembly quality is not measured with contiguity metrics alone—whether scaffold or contig N50. We now acknowledge this more clearly in the manuscript and have revised both our filtering strategy and manuscript text accordingly.

First, regarding sequencing technology, we have now included long-read (ONT or PacBio) and short-read (Illumina) assembly as a predictor in the phylogenetic mixed models. Moreover, the majority of assemblies in our dataset were generated using long-read technologies (ONT or PacBio), most of them polished with Illumina reads. We have made this clearer in the Methods to avoid any ambiguity.

Second, we have revised our assembly-quality thresholds and now provide two alternative annotation datasets:

(i) a full dataset including all species, intended as a comprehensive gene-level resource.
(ii) a high-confidence subset restricted to assemblies meeting stricter criteria (BUSCO completeness >97%, contig N50 >2 Mb, and OMArk contamination <5%).

We included the full annotation set to maximise accessibility and utility of gene sequences across the family. Excluding lower-quality assemblies would remove many perfectly usable gene models, even in genomes where only a subset of genes are missing or fragmented. As mentioned above, many users may simply wish to retrieve and align a particular gene to examine conserved regions, motifs, or domains, and we did not want to make entire species unavailable for such purposes because other parts of the genome are incomplete.

We fully agree that heterogeneity in assembly quality poses a serious challenge for analyses that depend on accurate gene content, such as gene family turnover. However, this heterogeneity is expected to have a much smaller impact on sequence-based analyses such as dN/dS estimation, where genic regions are typically assembled well and downstream filtering is performed in a CDS-aware manner (using MACSE). Consistent with this, our analyses show only weak or marginal relationships between assembly contiguity and gene-level properties such as CDS length and gene number.

For these reasons, we choose to provide both the full annotation set and a stricter high-confidence subset. This allows users to select the dataset most appropriate for their specific questions while avoiding unnecessary exclusion of usable gene sequences. We believe this approach appropriately balances data quality concerns with the needs of different downstream analyses.

* Discussions of quality. L381-411

There are at least three discussion points here that seem ripe for reinterpreation. They probably also argue for minor reanalysis or exclusion of assemblies in some cases.

1) Confusion surrounding Drosophila pseudoobscura subspecies leads to inclusion of a bad assembly Short version: The authors included two Drosophila pseudoobscura assemblies when they should have included only the RefSeq assembly.

Longer version: In the manuscript, the authors comment on outliers in terms of CDS length. They highlight problems with one D. pseudoobscura assembly, noting the unusually short CDS length of GCA_000001765.3. However, in the methods, the authors suggest two relevant criteria by which they selected genomes, including RefSeq genomes and genomes from Kim et al. 2024. In both RefSeq and Kim et al. 2024, the only Drosophila pseudoobscura genome is GCF_009870125.1. In Table S4, the authors report two different D. pseudoobscura genomes (corresponding to the two accessions above). This is extremely confusing. As it happens, both are samples from Mesa Verde and are therefore not only both North American, but both are from the same locale. Obviously, neither is D. pseudoobscura bogotana, since that would have to be from Bogota. So, I don't understand what distinction the authors think they are making by calling one D. pseudoobscura and D. pseudoobcura ssp. pseudoobscura. Moreover, the nomenclature is internally contradictory and inconsistent in the supplemental table. Compare columns A-D in Table S4:

Drosophila_pseudoobscura_pseudoobscura 46245	Dpseudoobsc	Drosophila_pseudoobscura_pseudoobscura.GCA_000001765.3.rm.fna

Drosophila_pseudoobscura	7237	Dpseudoobscpseudoobsc	Drosophila_pseudoobscura.GCF_009870125.1.rm.fna

to the text:

"The mean CDS length for most species (296 of the 304 species) ranged between 1.42 and 1.74 Kbp. The outliers included Drosophila pseudoobscura ssp. pseudoobscura (GenBank

accession: GCA_000001765.3), which exhibits unusually short CDSs (mean length of 1.15 Kbp) but a total gene count of 14,546 but close to the family median of 14,249 genes (Fig 1 and S4 Table). This observation raises the possibility that many gene models in such assemblies may be fragmented, incomplete, or represent short repetitive elements, possibly reflecting issues with assembly quality or annotation [79, 80]."

I'm familiar with the major high quality genome assemblies in Drosophila. Suffice it to say there is a reason why RefSeq did not select GCA_000001765.3 as the representative genome for the species. As a consequence, please drop the GCA_000001765.3 accession in favor of the RefSeq assembly (GCF_009870125).

We thank the reviewer for pointing out this issue and agree entirely with their assessment. The inclusion of both *Drosophila pseudoobscura* assemblies, and the associated nomenclature, was confusing and inconsistent with our stated genome selection criteria. We have now removed the GCA_000001765.3 assembly from the annotation set and all downstream analyses, and retained only the RefSeq representative genome (GCF_009870125.1). All references to unusually short CDS lengths in *D. pseudoobscura pseudoobscura* have been removed from the text, and Table S4 and related figures have been updated accordingly to ensure consistent naming and accession usage throughout the manuscript.

2) Inclusion of two Drosophila americana subspecies assemblies (GCA_030788265.1 and GCA_019972375.1). The subspecies distinction may actually be relevant here, but then again, maybe not (GCA_030788265.1 doesn't report the locale, so it's hard to even guess which subspecies it is). In any event, it seems to me that using a shaky subspecies distinction derived from Genbank records to split hairs between two assemblies isn't well justified. Here, the course of action is less clear than above as one assembly appears to be more contiguous (N50) while the other appears to be more complete (BUSCOs). I thought it relevant to bring this to the attention of the authors, though. I think the inclusion of subspecies is far less useful than species and so I encourage the authors to pick one assembly and go with it rather than include both under the current subspecies ambiguity. Personally speaking, for this type of project, I wouldn't even include two equally high quality assemblies per species, even if they happen to be from clearly distinct subspecies. Incomplete lineage sorting between good species is hard enough without throwing subspecies into the mix.

We agree with the reviewer that the inclusion of multiple *Drosophila americana* assemblies, particularly given subspecies ambiguity and incomplete provenance information, is not well justified in a comparative framework of this scale. To avoid introducing unnecessary complexity or potential confounding effects, we have therefore removed *Drosophila americana* from the dataset entirely.

3) The authors state:

"For Drosophila miranda it has previously been shown that there are occurrences of gene gain on its neo-Y chromosome"

Drosophila miranda's neo-Y has indeed acquired about 3,000 genes since its formation. Importantly, however, the Neo-Y resulted from a fusion between Muller element C (called Chromosome 3 in D. pseudoobscura) and the ancestral Y, forming a neo-Y and a neo-X. As it happens, Muller C has around 3,000 genes as well. Since this fusion happened so recently, the genes between the neo-Y and neo-X will appear to be duplicates through the lens of naive annotation approaches which rely on the assumption that chromosomes aren't homologs in the recent past. Since both are descendants of an autosomal Muller C, this assumption is violated. As it happens, if you subtract 3,000 duplicated genes from Mahajan and the 3,000 genes from a diverging pair of Muller C neo-sex chromosomes from the number of D. miranda genes, you get about 14,653 genes in D. miranda (assuming 20,653 genes from Table S4). This is extremely close to the mean of the genus (14,543, again according to Table S4). So, D.

miranda isn't all that anamalous after all once its biology is understood and accounted for. Might this also be true for other genomes in the dataset?

To confirm my intuition about whether the Genbank assembly analyzed by the authors actually contains the Neo-Y, I spent about 9 or 10 minutes doing a simple bioinformatics experiment:

1. download the D. miranda genome from GenBank 2. filter using bioawk to separate the NW contigs from the NC scaffolds ($name ~ /NC/ or $name ~ /NW/) and redirect them to different fasta files 3. upload them to D-Genies and inspect the dot plot. The striking result is that NC_046676.1 scaffold (representing the Neo-X) aligns to contigs NW_022881603.1 and NW_022881614.1, which correspond to the Neo-Y1 and Neo-Y2 scaffolds in Mahajan et al. 2018, respectively.

In conclusion, gene gain is almost certainly not the sole explanation for the duplications and increased number of annotated genes. Please confirm whether my quick and dirty inference that many of these duplicates are Neo-Y and Neo-X homologs. If so, then they aren't duplicates. They are simply diverging neo-sex chromosomes. Please correct the manuscript accordingly and see if this type of insight can be baked into the resource's curation.

A quick and dirty way of validating D. miranda's assembly in this context is to simply repeat the analysis after witholding the Neo-Y scaffolds, which should be almost exclusively classified as duplicates through naive homology approaches.

In Mahajan's original paper, the relevant numbers are:

"In total, we identified 6,448 genes on the neo-Y, and 3,253 genes on the neo-X, compared to 3,087 genes on the ancestral autosome that gave rise to the neo-sex chromosome."

We thank the reviewer for this detailed and insightful comment and fully agree with their interpretation. The elevated gene count and ‘apparent’ duplication signal observed in *Drosophila miranda* primarily reflect the recent fusion between Muller element C and the ancestral Y chromosome, resulting in homologous gene copies retained on the neo-X and neo-Y chromosomes, rather than widespread independent de novo gene duplication.

We confirm the reviewer’s inference that the GenBank assembly analysed here contains both neo-X and neo-Y scaffolds. As noted by the reviewer, scaffold NC_046676.1 (Chromosome 3) shows clear homology to contigs NW_022881603.1 and NW_022881614.1, corresponding to neo-Y1 and neo-Y2 as described in Mahajan et al. (2018). Consequently, many genes classified as duplicated by naive homology-based approaches are in fact homologous gene pairs derived from the same ancestral Muller C autosome. When these neo-sex homologs are accounted for, the inferred gene number for *D. miranda* falls very close to the genus mean, consistent with the reviewer’s calculation.

We have therefore revised the manuscript to correct our interpretation of *D. miranda* gene number and duplication. The text in the manuscript now states the apparent excess of genes in *D. miranda* is largely explained by retention of homologous gene copies on the neo-X and neo-Y following chromosome fusion, rather than true gene gain. Correspondingly, *D. miranda* is no longer presented as an anomalous outlier once its sex chromosome biology is taken into account.

With respect to incorporating this insight into the resource, we agree that *D. miranda* represents a clear and well-supported case where neo-sex chromosome evolution strongly influences gene content. We have therefore treated it as a case study in the manuscript and explicitly caution that gene-content metrics for this species should be interpreted in this biological context. While similar mechanisms could plausibly contribute to elevated gene counts in other species, current assemblies for most taxa lack sufficient resolution or annotation of sex-linked scaffolds to confidently identify neo-sex homologs in a systematic way. For this reason, we have not applied analogous corrections to other genomes, but instead discuss neo-sex chromosome evolution as an alternative biological explanation for apparent gene gain where appropriate.

* Figure 1: The violin plots are too numerous and individually take up too little of the chart to provide readily accessible information. It's a clever idea that I think fails in the actual execution.

We thank the reviewer for this helpful suggestion. To improve clarity and readability, we have replaced the violin plots with tile layers and now summarise mean CDS length, BUSCO completeness, genome size, and contig N50.

* "we first gathered available RNA-seq data to provide transcript evidence that can help resolve ambiguities in gene."

Singular gene?

This has been corrected.
